# Focal brain cooling suppresses spreading depolarization and reduces endothelial nitric oxide synthase expression in rats

**DOI:** 10.1016/j.ibneur.2024.05.001

**Published:** 2024-05-12

**Authors:** Yuya Hirayama, Hiroyuki Kida, Takao Inoue, Kazutaka Sugimoto, Fumiaki Oka, Satoshi Shirao, Hirochika Imoto, Sadahiro Nomura, Michiyasu Suzuki

**Affiliations:** a Department of Neurosurgery, Graduate School of Medicine, Yamaguchi University, Japan; b Department of Physiology, Graduate School of Medicine, Yamaguchi University, Japan; c Organization of Research Initiatives, Yamaguchi University, Japan

**Keywords:** multimodal recording, focal brain cooling, eNOS, KCl, spreading depolarization, spreading hyperemia

## Abstract

This study aimed to investigate the effects of focal brain cooling (FBC) on spreading depolarization (SD), which is associated with several neurological disorders. Although it has been studied from various aspects, no medication has been developed that can effectively control SD. As FBC can reduce neuronal damage and promote functional recovery in pathological conditions such as epilepsy, cerebral ischemia, and traumatic brain injury, it may also potentially suppress the onset and progression of SD. We created an experimental rat model of SD by administering 1 M potassium chloride (KCl) to the cortical surface. Changes in neuronal and vascular modalities were evaluated using multimodal recording, which simultaneously recorded brain temperature (BrT), wide range electrocorticogram, and two-dimensional cerebral blood flow. The rats were divided into two groups (cooling [CL] and non-cooling [NC]). Warm or cold saline was perfused on the surface of one hemisphere to maintain BrT at 37°C or 15°C in the NC and CL groups, respectively. Western blot analysis was performed to determine the effects of FBC on endothelial nitric oxide synthase (eNOS) expression. In the NC group, KCl administration triggered repetitive SDs (mean frequency = 11.57/h). In the CL group, FBC increased the duration of all KCl-induced events and gradually reduced their frequency. Additionally, eNOS expression decreased in the cooled brain regions compared to the non-cooled contralateral hemisphere. The results obtained by multimodal recording suggest that FBC suppresses SD and decreases eNOS expression. This study may contribute to developing new treatments for SD and related neurological disorders.

## Highlights

• SD induced by administration of potassium shows multimodal phenomenon.
• Brain surface cooling at 15°C reduces KCl-induced SD.
• Hyperemia induced by SD is suppressed by FBC.
• SD suppression is associated with the suppression of eNOS expression.

## Graphical Abstract

**Figure:**
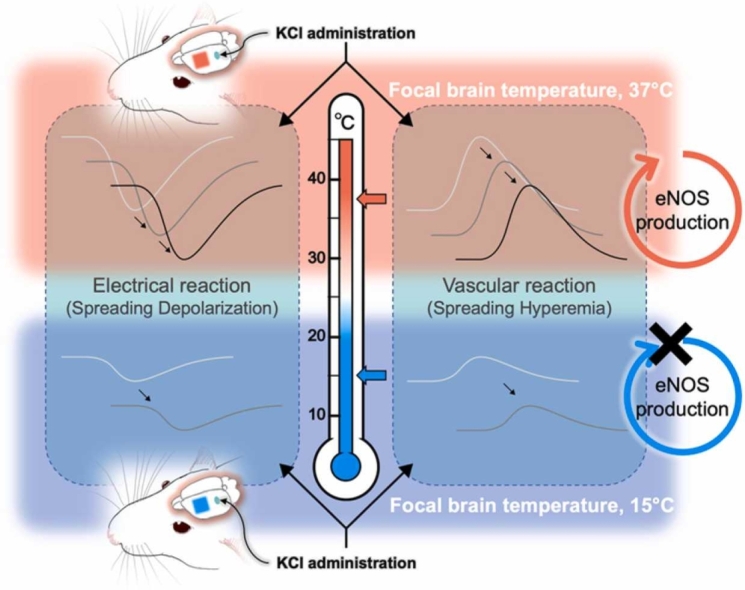

## Introduction

Spreading depolarization (SD) is a phenomenon characterized by waves of neuronal depolarization that travel across the brain tissue, causing transient changes in ionic gradients, leading to suppression of neuronal activity. This phenomenon is implicated in a variety of neurological disorders. SD propagates from a specific cerebral region with neuronal suppression and ischemia/hyperemia at a rate of 1.7–9.2 mm/min in gray matter (Dreier, 2011, Leao, 1944),. High extracellular potassium concentrations affect astrocytes (Seidel et al., 2016), neurons (Leao, 1944), and vascular functions (Dreier, 2011), triggering SD and leading to changes in cerebral blood flow (CBF), including spreading hyperemia (SH) and oligemia under physiological conditions. Typically, these are neurovascular responses caused by vasodilation and increased CBF, but the electroencephalographic amplitude is depressed during SD (Dreier, 2011). SD is potent and closely related to complex diseases, such as aura in migraine, and is involved with delayed cerebral ischemia in severe stroke (Lauritzen et al., 2011). Substances that suppress SD have been developed, and most have been shown to modulate specific characteristics of SD; however, only few have succeeded in significantly suppressing SD (Klass et al., 2018).

Recently, the efficacy of therapeutic hypothermia has been investigated for the acute treatment of severe subarachnoid hemorrhage and traumatic brain injury as well as in the treatment of status epilepticus (Dietrich and Bramlett, 2017, Kuczynski et al., 2020). Therapeutic hypothermia is one of the most potent neuroprotective strategies. However, the current literature indicates many challenges in successfully utilizing therapeutic hypothermia in patients with cerebral injuries. These include difficulties in effectively managing temperature, adverse effects (e.g., increased risk of infection), and variable responses in individual patients. Although several studies have tried to address these challenges, they remain to be solved. Recent studies suggest that therapeutic hypothermia reduces microvascular spasms and delayed cerebral ischemia, leading to improved functional outcomes (Kuramatsu et al., 2015). Yenari et al. (2000) found that transient apparent diffusion coefficient changes related to SD are prolonged in mild hypothermia compared with normothermia. The finding that mild hypothermia inhibits or slows the propagation of SD-related activity suggests that it also reduces metabolic burden to the brain. Interestingly, Bures et al. (1957) demonstrated that systemic cooling rats from 33.7°C to 18.8°C prolongs and slows SD, indicating similar benefits in deep hypothermia. Recent studies continue to explore the neuroprotective effects of therapeutic hypothermia, with findings such as those by Kentar et al. (2023) demonstrating that 32°C mild hypothermia can significantly reduce the frequency and expansion of SDs, as well as infarct size in a swine model of ischemic stroke. Regardless, the effects of 30°C hypothermia on the propagation of peri-infarct depolarizations with temporal and spatial resolutions using nicotinamide adenine dinucleotide fluorescence images delayed the appearance of peri-infarct depolarizations; however, it did not suppress the occurrence of peri-infarct depolarizations (Sasaki et al., 2009). Currently, the therapeutic effects of therapeutic hypothermia on suppressing SD remain controversial (Dietrich and Bramlett, 2017).

Focal brain cooling (FBC) is a well-established method for suppressing epileptic discharges from epileptogenic foci. Our previous (Fujii et al., 2012, Imoto et al., 2006, Inoue et al., 2017, Kida et al., 2012, Oku et al., 2009) and other (Rothman, 2009, Yang and Rothman, 2001) studies have shown that FBC at 13–15°C using a Peltier chip or a temperature-controlled metal plate can suppress epileptic discharges induced by cerebral infusion of convulsant drugs without causing histological damage and cortical dysfunction in a wide range of animals. Interestingly, the mechanism of SD is closely related to epileptic discharges (COSBID study group, 2012, Rogawski, 2012, Wei et al., 2014). This suggests that the suppressive effect of hypothermia on SD may also be similar for epileptic discharges and raises the possibility that FBC can control not only hyperactivity, as in epileptic discharge, but also complex activities that cause simultaneous hyperactivity and hypoactivity, as in SD.

Nitric oxide (NO), a signaling molecule that plays a pivotal role in various physiological processes such as neurotransmission and vasodilation, is key in neurovascular dynamics. NO is synthesized by the nitric oxide synthase (NOS) enzyme family, which includes neuronal NOS (nNOS), inducible NOS (iNOS), and endothelial NOS (eNOS). While nNOS is primarily involved in neuronal signaling and iNOS is generally upregulated during inflammation, eNOS is predominantly found in endothelial cells and is crucial for regulating vascular tone. Further, previous research has indicated that eNOS plays a significant role in cerebral blood flow regulation and is implicated in various neurological disorders, such as stroke (Zhu et al., 2016) and migraine (Pradhan et al., 2018). Thus, given its critical role in vascular regulation and its implication in neurological disorders, in this study, we aimed to investigate how FBC influences eNOS expression and its subsequent impact on SD and related vascular responses.

Our study aimed to examine the impact of FBC on SD propagation, focusing on the activity of neurons and vascular functions related to SD. This study presents the effects of brain cooling on SD based on multimodal electrophysiological recording and Western blot analysis of the cortical surface in rats. This study is expected to provide valuable insights into the neuroprotective potential of FBC after brain injury and contribute to developing of new therapies for SD-related neurological diseases.

## Materials and methods

### Animals

Healthy adult male Sprague–Dawley rats (300–360 g, 10–14 weeks old, male; Kyodo Co., Ltd., Fukuoka, Japan) housed in a temperature and humidity-controlled room (22.0°C ± 2.0°C, 45% ± 5%) were provided with *ad libitum* access to specific feed and water. The sample size selection for the electrophysiological and molecular study (n = 16 and n = 24, respectively) was substantiated by prior work by Sugimoto et al. (2018), which investigated the impact of Cilostazol on the duration of spreading depolarization and ischemia using a similar experimental setup. Said study provided insights into SD with high statistical significance, and its experimental design bears high relevance to our research. Animal experiments were performed using protocols approved by the Institutional Animal Care Committee at Yamaguchi University School of Medicine.

### Surgical procedure and SD model

**Fig. 1:**
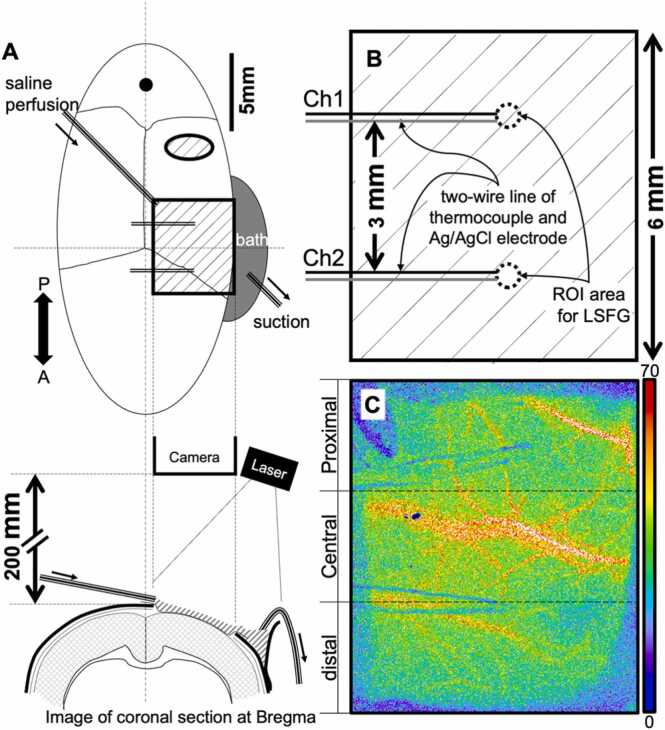
(A) Schema demonstrates the locations of the burr hole (oval with diagonal lines), cranial window (rectangle with diagonal lines), thermocouples and ECoG electrodes, inflow and outflow tube, and equipment (camera and laser emitter) for Laser speckle flow imaging (LSFI). An inflow (perfusion) and outflow (suction) tube allowed the temperature of the cortex to be controlled with saline. Parafilm covered the brain surface to avoid direct contact with saline. This was also accompanied by immobilization of the sensor position. In addition, a cover glass, curved to fit the shape of the head, was placed over the entire brain surface to distribute the saline solution. CBF was measured by the LSFI technique from the overall area of the cranial window. Potassium chloride (KCl) solution was administrated into the burr hole. (B) An enlarged view of the cranial window. Region-of-interest was set at the tip of the wire lines to analyze the regional CBF. (C) A two-dimensional map of the CBF recorded over the cranial window during the incubation period. The two thin blue lines indicate the artifact affected by the thermocouples and ECoG electrodes.

Rats were first anesthetized with urethane (1.25 g/kg, i.p.). During surgery, the body temperature (BoT) was obtained using a thermocouple (RET-2, Physitemp Instruments Inc., Clifton, NJ, USA) and kept constant at 37.5°C ± 0.5°C. The left femoral artery was cannulated to monitor the arterial blood pressure and continuously obtain blood samples. The skull was fixed using a stereotactic apparatus (SR-6 N; Narishige Group Inc., Tokyo, Japan), and skin incision was performed. One cranial window and one burr hole were created using a dental drill over the ipsilateral area (cranial window: L1.0–5.0 mm, A3.0 mm, P3.0 mm, burr hole: center, P7.0 mm, L4.0 mm, width, 4.0 mm, height, 1.0 mm) (Fig. 1). The dura was then carefully removed, then two Ag/AgCl wire electrodes (tip diameter = 0.08 μm, Unique Medical Co., Ltd. Tokyo, Japan) and two Teflon coated thin thermocouples (tip diameter = 0.23 μm, IT-24 P; Physitemp Instruments, Inc., Clifton, NJ, USA) were set on the brain surface at the distal (1.5 mm anterior and 3.0 mm lateral to the bregma) and proximal (1.5 mm posterior and 3.0 mm lateral to the bregma) portions of the cranial window. The brain surface was covered with thinly spread Parafilm to fix these sensors and to protect the brain surface against the temperature-controlled perfusion solution. We created another burr hole on the cerebellum to set the reference Ag/AgCl electrode. Finally, the cranial window was covered by a specially curved slide glass. Cerebrospinal fluid drainage was used to manage brain swelling associated with opening the dura. A cannulation hole was created on the skull (over the cisterna magna). A midline incision was made in the neck along the occipital bone until the atlanto-occipital membrane was visible. A micro-incision was then made with a needle in the cisterna magna. Leaking spinal fluid was systematically vacuumed. Detailed images of the experimental setup can be found in Supplementary Figure 1.

**Fig. 2:**
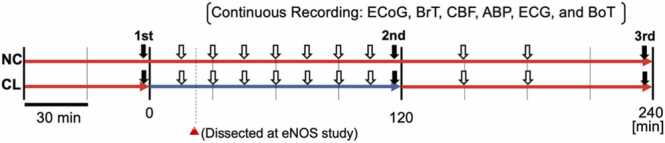
Experimental protocols of the study. The black arrow indicates the analysis point of arterial blood gas and pH (total three-time points). The white arrow indicates the period of Potassium chloride (KCl) administration (total of 9 periods). The blue line indicates the cooling period in the cooling (CL) group.

To induce SD, we initially intended to replicate the methodology employed by Ayata et al. (2006). A cotton ball (2 mm in diameter) soaked in 1 M potassium chloride (KCl) was placed on the pial surface of the burr hole and maintained moist by placing 5 μL of KCl (1 M) solution every 15 min to ensure continuous SD. The number of KCl-induced SD and related events was observed for 2 h from the cooling onset (Fig. 2). After 120 min of cooling, we tested if rewarming could induce SD. However, cooling has transient cooling effect that persist, even after the start of the rewarming period (Nomura et al., 2014). Therefore, we attempted to induce SD by KCl on two separate occasions, 30 and 60 min after rewarming initiation.

Each experiment was terminated by KCl administration into the femoral artery under deep anesthesia, and we confirmed the biological zero of laser speckle value upon cardiac arrest.

### Multimodal recording and FBC

In this experiment, KCl-induced SD and related events were measured using multimodal recording techniques that also recorded BrT, alternating current range of electrocorticography (AC-ECoG), direct current range of electrocorticography (DC-ECoG), and CBF according to the procedure used by Sugimoto et al. (2018) (Fig. 2). Multimodal recording promptly detects electrophysiological and neurovascular response on the brain surface simultaneously and provides valuable information on the neurophysiology underpinning phenomena (Makarenko et al., 2016, Vespa et al., 2014). BrT and ECoG were amplified with a thermometer (100 A; Unique Medical Co., Ltd., Japan) and a differential extracellular amplifier (Model 4002 EX1; Dagan Co., Minneapolis, MN, USA), respectively. The laser speckle flow imaging (LSFI) technique is the most valuable method to detect CBF changes on the brain surface. We recorded the two-dimensional CBF changes through the cranial window with a sample rate of 1 Hz (OZ-2; Omegawave, Inc., Japan). Arterial blood pressure, heart rate, BoT, CBF, BrT, and wide-band ECoG were recorded continuously, while arterial blood gasses and pH were measured thrice during non-cooling (NC), cooling (CL), and rewarming. All data were digitized at 2 kHz at 16-bit resolution using a multichannel recorder (PowerLab 8/35; ADInstruments Pty. Ltd., Bella Vista, NSW, Australia) and stored on a PC. Offline and online analyses were performed using LabChart software (LabChart8; ADInstruments, Pty. Ltd., Bella Vista, NSW, Australia).

Warm or cold saline was perfused on the surface of one hemisphere to maintain BrT at 38°C or 15°C in the NC and CL groups, respectively. Cooling the brain at 15°C can suppress abnormal brain activity while maintaining healthy brain function (Fujii et al., 2012, Kida et al., 2012). This temperature has been established as the “optimal temperature” that effectively treats epileptic seizures, but it is also expected to impact other diseases (He et al., 2013). Therefore, in the CL group, cooling was started to lower the BrT to 15°C before KCl administration. The cooling rate was carefully controlled to ensure that the BrT dropped to 15°C before inducing spreading SD with KCl (Supplementary Figure 2). Brain temperature quickly fell to 17.8°C within one minute of starting the cooling process and further decreased to 15°C within five minutes. The cooling process followed a sigmoidal curve, with the fastest rate of temperature decrease recorded at −7.19°C per second, 19 seconds after the cooling began.

### Evaluation of KCl-induced SD and related events

We performed signal treatments before analysis to remove excess noise and extract the necessary information. The ECoG was divided into AC and DC-ECoG using a digital filter with a 0.1–50-Hz band-pass and a 0.1-Hz low-pass, respectively. The BrT and BoT were smoothed with a moving average (1-s window). CBF changes were measured by a region-of-interest analysis on two regions. region 1 (proximal) and region 2 (distal) were set near the tip of each ECoG electrode with a diameter of 0.5 mm (See Fig. 1). Spike-like large-amplitude artifacts were removed from recorded CBF, BrT, and wide-band ECoG, and a short segment was interpolated instead.

For each treated signal, we performed analysis using the following methods. First, the peak CBF, the trough of DC-ECoG, and the Root Mean Square (RMS) of AC-ECoG were estimated to determine the changes from their baseline values. Second, each KCl-induced SD and related event was evaluated and annotated by three persons. If there was inconsistency among the observations, another person made the final judgment. Third, each modality’s duration of KCl-induced SD and related event was estimated as the period until the recovery rate became minimal. Fourth, the propagation speed of SD and SH was calculated based on the time and distance between the two recording sites. Finally, the mean value was calculated from the first through the seventh KCl-induced SD and related events to obtain the data for one individual, which was used to calculate the mean value for each group.

### eNOS study

eNOS is involved in important signaling mechanisms in the cerebrovascular system that can generate a local NO pool, leading to NO production by phosphorylation and control of CBF (Yamada et al., 2000). To determine the influence of FBC on SH, we performed Western blot analysis of eNOS.

When SH reached the center of the cranial window after the first KCl administration using LSFI, the rat was beheaded, and the brain was divided into three coronal sections of 2.0-mm width in the cranial window (distal, central, and proximal from burr hole). With the expectation that the timing of depolarization wave arrival is essential to determine the effect of SD on eNOS expression and activation, we divided the evaluation site into three areas to avoid the dilution of the impact. We sliced brain tissue containing surface layers (layers I–III), and tissues were incubated for 3 min in ice-cold buffer containing 0.32 M sucrose and 20 mM Tris-HCl (pH 7.5). Dissected cortical tissues were homogenized in 200 μL of buffer containing 50 mM Tris-HCl (pH 7.4), 0.5% Triton X-100, 0.5 M NaCl, 10 mM ethylene diamine tetraacetic acid (EDTA), 4 mM glycol ether diamine tetraacetic acid (EGTA), 1 mM Na_3_VO_4_, 50 mM NaF, 40 mM sodium pyrophosphate, 1 mM protease inhibitor, and 1 mM Dithiothreitol (DTT). Insoluble material was removed by 10-min centrifugation at 15,000 rpm. Samples containing equivalent amounts of protein based on BCA analysis (Thermo Scientific, Rockford, IL, USA) were heated at 100°C for 3 min in Laemmli sample buffer and subjected to SDS-PAGE for 30 min at 200 V. Proteins were transferred to an Immobilon PVDF membrane for 1 h at 100 V. Membranes were blocked for 1 h at room temperature in T-TBS solution containing 50 mM Tris-HCl (pH 7.5), 150 mM NaCl, 0.1% Tween 20, and 5% skim milk followed by overnight incubation at 4°C with anti-eNOS (1:1000; BD Biosciences, Tokyo, Japan), anti-phospho-eNOS (Ser1177) (1:1000; Cell signaling), and anti-β-Tubulin (1:1000; BioLegend, San Diego, CA, USA). This step was followed by incubation with HRP-conjugated goat anti-rabbit IgG for phosphorylated-eNOS (p-eNOS) and β-Tubulin (1:5000, Millipore) and HRP-conjugated goat anti-mouse IgG (1:5000, Millipore) for eNOS. Bound antibodies were visualized using Amersham ECL Western Blotting Detection Reagents (GE Healthcare, Chalfont St. Giles, UK) and semi-quantitatively analyzed using the Image-J program (National Institutes of Health, Bethesda, Maryland, USA).

In addition, experiments under no-KCl conditions were conducted in the same manner as above to examine the stability of tubulin to cooling. However, since SD did not induce, animals were decapitated, assuming a hypothetical SD timing.

### Statistical analysis

All data were processed using Excel (Microsoft Corporation, Redmond, WA, USA) or MATLAB (Mathworks, Inc., Natick, Massachusetts, USA), and we used JMP (SAS Institute Inc., Cary, North Carolina, USA) for statistical tests. We applied the RMS method to the measured data to validate the amplitude changes of the spreading depression. The analysis used the sliding window method of 10 s to allow RMS values to be calculated for each segment of the signal. Statistical analyses of physiological parameters were carried out using the Friedman test followed by Wilcoxon signed rank test. Statistical comparison of the two groups was performed with the Mann–Whitney U test for electrophysiological data. In the analysis of eNOS data, we performed either a 2-way or 3-way analysis of variance (ANOVA). The analysis included the following three factors: ‘Group’ (Factor 1) was categorized into two levels - CL and NC. The CL group included rats subjected to FBC, while the NC group served as control. ‘Hemisphere’ (Factor 2) corresponds to the hemispheric side of the brain, with two levels - Left and Right. Left corresponded to the side of the brain directly exposed to the temperature control, while the Right served as naive control (non-craniotomized and non-temperature-controlled). ‘Dissected Region' (Factor 3) divided the brain into three regions based on proximity to the site of KCl application and temperature-control: Proximal, Central, and Distal. These regions were intended to capture potential regional differences in response to FBC and SD. If the main effect in the 2-way ANOVA was significant, a post-hoc test was performed using the Wilcoxon signed-rank test with Bonferroni correction. If the interaction was significant, we used Tukey's HSD test. In addition, we employed Tukey's HSD test for post-hoc tests after the three-way ANOVA. In the description of the average data, the standard deviation and standard error of the mean were used to explain the data distribution. P-values of <0.05 was considered statistically significant.

## Results

### Effects of FBC on electrical and physiological responses to potassium chloride (KCl) application

To evaluate the physiological status under different conditions, we summarized the serial changes in physiological parameters in Table 1. This serves as a baseline for understanding how cooling affects the overall physiology. In the analysis, one rat in the NC group was excluded because of a technical problem with acquiring multimodal recording data. Throughout the experiment, the mean arterial blood pressure remained within physiological limits, although there was a significant difference between the first and second sampled points in the CL group. (P < 0.05, Friedman test followed by Wilcoxon signed rank test, Table 1). Blood gas and pH were mostly within physiological limits, although there was a significant difference only in the CL group. Continuous FBC increased PCO_2_ levels (P < 0.05), and pH slightly increased at the end of the rewarming period (P < 0.05).

**Table 1:** Serial changes in physiological parameters.

Group	Sampled point	HR [bpm]	MABP [mmHg]	BoT [°C]	pH	BrT [°C]		Blood gas [mmHg]	
						Ch1	Ch2	PO_2_	PCO_2_
NC (n = 7)	1st	397.9 ± 4.09	76.02 ± 3.08	37.44± 0.09	7.4 ± 0.01	37.38 ± 0.13	37.75 ± 0.11	93.56 ± 7.39	37.13 ± 0.97
	2nd	395.70 ± 5.90	78.99 ± 2.81	37.47 ± 0.07	7.43 ± 0.02	37.35 ± 0.13	37.76 ± 0.12	94.24 ± 5.38	34.60 ± 1.62
	3rd	410.12 ± 11.30	82.35 ± 3.82	37.53 ± 0.07	7.44 ± 0.01	37.49 ± 0.13	37.87 ± 0.13	95.4 ± 5.37	32.36 ± 1.72
CL (n = 8)	1st	397.33 ± 6.86	75.58 ± 3.65	37.50 ± 0.08	7.43 ± 0.01	37.29 ± 0.08	37.77 ± 0.12	87.85 ± 2.13	34.43 ± 0.84
	2nd	397.68 ± 6.81	80.02 ± 3.21 *	37.44 ± 0.10	7.39 ± 0.02 #	15.38 ± 0.18 * #	14.85 ± 0.19 * #	90.61 ± 1.10	39.54 ± 0.67 *
	3rd	401.23 ± 9.88	83.21 ± 4.85	37.57 ± 0.07	7.42 ± 0.02	37.25 ± 0.09	37.70 ± 0.15	88.80 ± 1.61	35.29 ± 1.12

There were significant differences in the Freidman test followed by Wilcoxon signed rank test with Bonferroni correction in the CL group. * P < 0.05 vs. 1st sampled point. # P < 0.05 vs. 3rd sampled point. Values are mean ± standard deviation. NC = non-cooling, CL = cooling, HR = heart rate, MABP = mean arterial blood pressure, BrT = brain temperature, BoT = body temperature, PO 2 = arterial oxygen tension, and PCO 2 = arterial carbon dioxide tension. Ch1 and Ch2 are the proximal and distal channels of the thermocouple from the potassium chloride (KCl) application point (burr hole). The three sampled points correspond to the end of the NC, CL, and rewarming period as shown in Fig. 2, respectively.

**Fig. 3:**
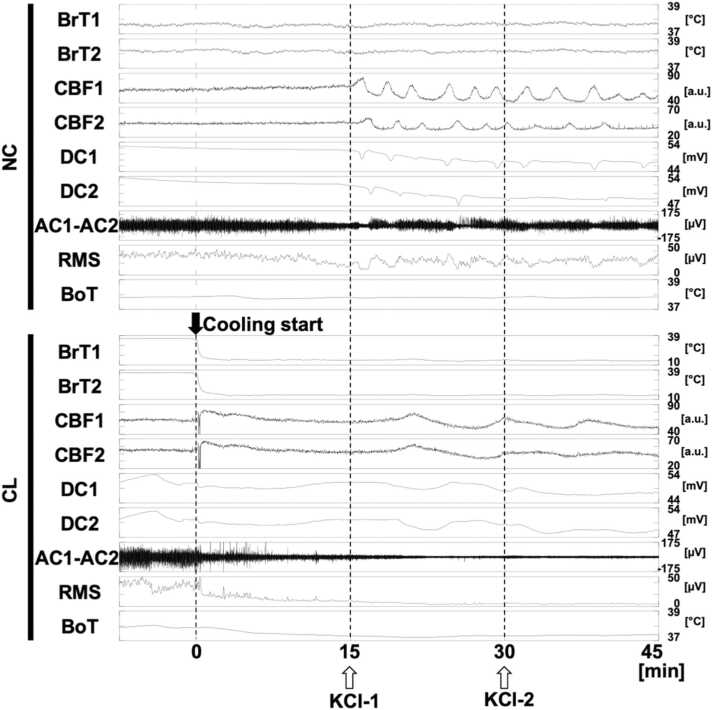
Representative tracing of the multimodal signals obtained in both non-cooling (NC) and cooling (CL) groups upon cooling and the first two periods of Potassium chloride (KCl) administration. DC1 and DC2 are ch1 and ch2 of DC-ECoG, respectively, and filtered by a low-pass filter (<0.1 Hz). AC1–AC2 are differential recordings between ch1 and ch2 of AC-ECoG filtered by a band-pass filter (1–50 Hz). RMS is calculated by differential signals between AC1 and AC2. The values of CBF1 and 2 are in arbitrary units (a.u.).

To further investigate into the effects of FBC, we administered KCl directly to the cortex. This triggered SD, SH, and spreading depression, as shown in Fig. 3 [upper] and Supplementary Movie 1. This finding is significant because it highlights the influence of FBC on multiple electrophysiological variables. Additionally, two-dimensional CBF changes were observed throughout the experiments. These events should occur in the coupling, but in the present study, there were cases where they could not be captured as coupling events with the expectation that the sensor’s sensitivity was affected.

The following is the Supplementary material related to this article Video S1

Video S1

Interestingly, we observed that FBC led to an obscuration of SD waveforms and a more complex propagation path. This suggests that FBC may interfere with the normal dynamics of SD. (Fig. 3 [lower], Supplementary Movie 2). For example, FBC suppressed SH, leading to turn around behavior at the brain surface instead of propagating caudally from the proximal side.

The following is the Supplementary material related to this article Video S2

Video S2

**Fig. 4:**
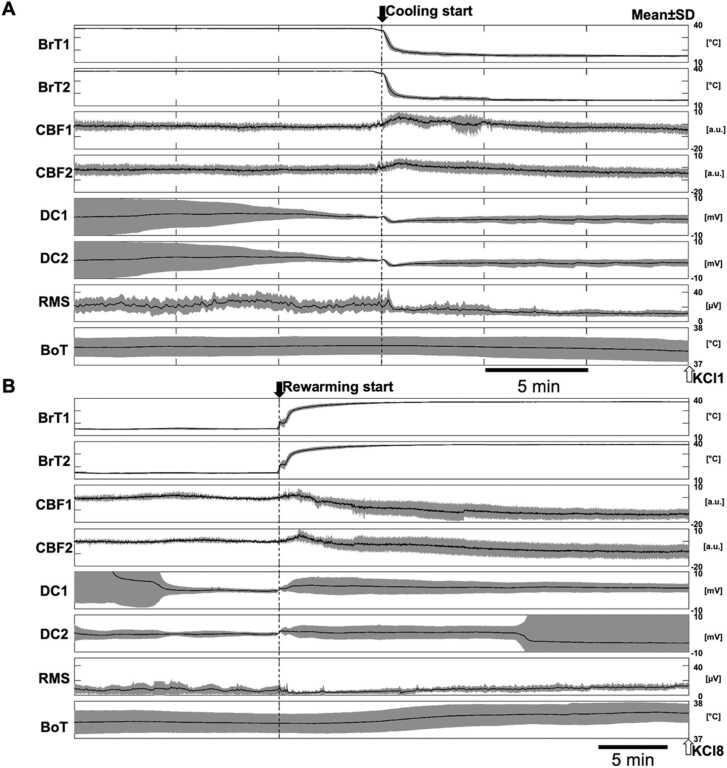
Average activity of multimodal signals A) before and during cooling and B) before and during rewarming in the cooling (CL) group. Solid lines and gray-filled areas are the mean ± standard deviation values, respectively. DC1 and DC2 are DC-ECoG filtered by a low-pass filter (< 0.1 Hz). Because baseline DC-ECoG and CBF were different for each measurement, we zero-corrected with respect to the time of cooling initiation. Root mean square (RMS) is calculated by differential signals between AC-ECoG1 and AC-ECoG2. The values of CBF1 and 2 are in arbitrary units (a.u.).

The average response of multimodal signals to FBC in the eight rats in the CL group is shown in Fig. 4. The response of each modality before and after cooling is shown in Fig. 4A. Multimodal brain signals were affected by the decreases in brain temperature (BrT). The baseline voltage of the DC-ECoG was temporarily decreased, and the root mean square (RMS) amplitude of AC-ECoG was sharply and continuously reduced. CBF exhibited a transient increase immediately after the start of cooling and a mildly declined baseline level. Upon rewarming (Fig. 4B), neither DC-ECoG nor AC-ECoG recovered immediately, but fluctuations, which appeared to be a physiological response, were gradually observed. By contrast, contrary to our expectations, CBF showed a slow decrease. Prolonged cooling may have led to a condition wherein there was difficulty in the immediate resumption of neural activity.

Given these findings, our strategy for multimodal measurements involved analyzing each event separately, regardless of the method used for measurement. This approach was necessary to disentangle the complex effects of FBC on SD and related phenomena. Additionally, we excluded a detailed evaluation of the rewarming period for the exact relationship between FBC and SD.

### Detailed effect of FBC on KCl-induced SD and related events

**Fig. 5:**
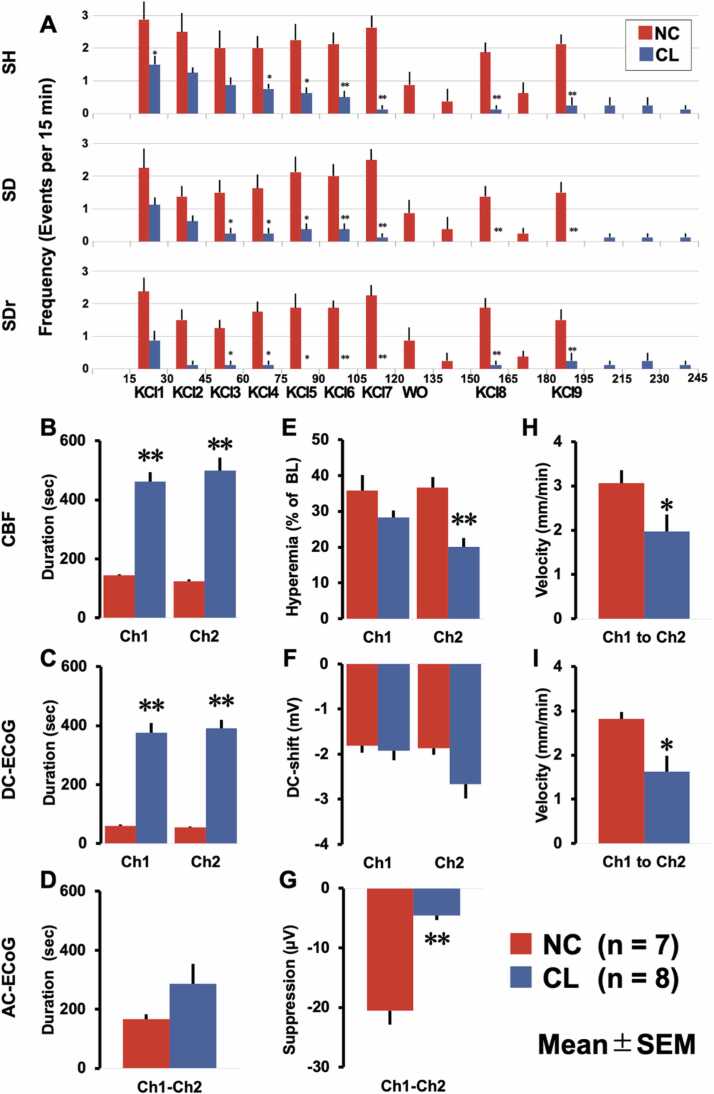
(A) Potassium chloride (KCl)-induced events in each KCl administration period. Time-interval histograms indicate the frequency of occurrence of SH (spreading hyperemia), SD (spreading depolarization), and SDr (spreading depression) every 15 min that coincides with each group’s interval of KCl administration. (B-I) FBC-induced relaxation compared with non-cooling (NC). FBC delayed the duration, suppressed the amplitude, and slowed the velocity in all modalities. The 3 ×3 matrix indicates the relationship among the three modalities of brain activity; first to third rows are CBF, DC-ECoG, and AC-ECoG, while first to third columns are duration, amplitude change, and velocity, respectively. a* P < 0.05, ** P < 0.01. Error bars indicate SEM. WO = wash out, BL = baseline.

To dissect the time-dependent effects of FBC on SD, Fig. 5 A provides a bar chart that breaks down the frequency of KCl-induced SD and related events in 15-minute intervals, coinciding with each group's KCl administration schedule. In the NC group, KCl-induced SD and related events showed a similar frequency of occurrence compared with that in a previous study (Ayata et al., 2006). Contrastingly, in the CL group, we noticed a gradual decline and eventual disappearance in the frequency of KCl-induced SD and related events. In particular, spreading depression disappeared more rapidly and completely than other events due to the strong suppression of AC-ECoG amplitude by cooling. This phenomenon resembles isoelectric SD, where the clustering of SD episodes leads to the disappearance of spreading depression (Major et al., 2020). Nevertheless, SD induced during the cooling state is deemed to represent a fundamentally different attribute, characterized by the elongation of the DC shift's profile. This stark difference highlights the pivotal role that FBC may play in modulating these neural activities. Unfortunately, due to prolonged cooling (2 h), KCl-induced SD and related events were not sufficiently induced despite administration of KCl twice during the rewarming period (Fig. 5). Thus, we focused and elaborated on the following observations during the cooling period. All analytical results of the derived electrophysiological experiment are shown in Fig. 5B–I, which is denoted as a matrix layout. FBC significantly increased the duration of hyperemia and negative DC-shift, except for the RMS of AC-ECoG (P < 0.05, Mann–Whitney U test, Fig. 5B–D). Fig. 5E-G show the amplitude of a negative DC-shift, the highest level of hyperemia, and spreading depression. FBC inhibited the amplitude change of two modalities of hyperemia and depression of AC-ECoG (P < 0.01; Figs. 5E and 5 G). Although not significant, FBC showed a tendency to increase the amplitude of negative DC-shift in ch2 (P < 0.1; Fig. 5 F). Importantly, we also observed reduced SH and SD propagation speed between channels ch1 and ch2 under cooling conditions. This suggests that FBC affects the frequency and dynamics of SD propagation (P < 0.05, Figs. 5H and 5I). Our results indicate that FBC significantly alters the occurrence and dynamics of KCl-induced SD and related events. These findings are crucial for understanding FBC's potential in controlling SD and its related pathologies.

### eNOS and p-eNOS expression during KCl-induced hyperemia

**Fig. 6:**
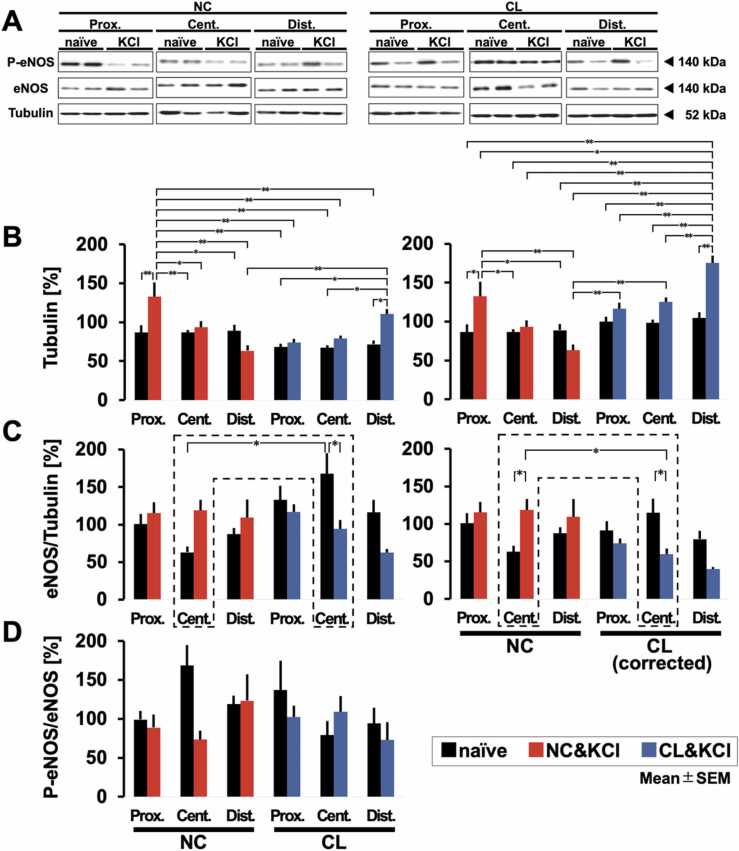
(A) Superficial cortical layers were dissected and analyzed by Western blot. Molecular masses of standards are indicated in kDa on the right. (B) Effect of cooling on tubulin during SD development. No correction (left) and correction (right) for the cooling-induced decrease in β-tubulin. (C) Total endothelial nitric oxide synthase (eNOS) was normalized to total β-tubulin. Potassium chloride (KCl) significantly decreased eNOS expression in the central region. No correction (left) and correction (right) for cooling-induced decrease in β-tubulin. (D) The phosphospecific signal of p-Ser1177 eNOS was normalized to total eNOS. * P < 0.05, ** P < 0.01. Error bars indicate SEM. Prox. = proximal, Cent. = Central, Dist. = Distal.

To further elucidate the molecular mechanisms underlying SH, particularly focusing on the role of eNOS, we conducted Western blot analysis with (Fig. 6 A) or without (Supplementary Figure 2) KCl application.

#### Tubulin and eNOS/tubulin in the no-KCl study

To confirm the stability of tubulin as a housekeeping protein, the effect of cooling on tubulin expression was investigated. Cooling led to suppression of tubulin expression (Supplementary Figure 2B). A 2-way ANOVA revealed a significant main effect for 'Group' (F(1,20) = 3.7911, P < 0.01). Further, post-hoc tests showed significant differences between CL and NC in the naive hemisphere (P < 0.05) and between CL and NC in the exposed hemisphere (P < 0.05). Thus, the effect of temperature propagated to the right hemisphere, which was untreated in the current study. Although the reason remains unclear, the temperature control of one side of the brain may have affected the other due to the small brain volume.

Correction coefficients were calculated to compensate for the effects of cooling on tubulin expression (Supplementary Figure 2D). For the naive hemisphere, the correction coefficient was 0.6323, while for the exposed hemisphere, the coefficient was 0.6845. Equivalence was ensured by applying these correction coefficients to the tubulin data. No significant effect was observed when eNOS/tubulin was calculated using corrected tubulin values (Fig. 2C). These correction coefficients were also employed in experiments inducing SD with KCl, as described below.

#### Tubulin in the KCl study

The effect of KCl-induced SD during BrT control on tubulin expression was affected, as shown below (Fig. 6B [left]). A 3-way ANOVA showed significant main effects for the factor 'Group' (F(1, 60) = 9.60, P < 0.01) and for 'Hemisphere' (F(1, 60) = 8.83, P < 0.01). Additionally, a significant interaction was found between 'Group' and 'Dissected region' (F(2, 60) = 11.97, P < 0.0001), as well as among 'Group,' 'Hemisphere,' and 'Dissected region' (F(2, 60) = 11.41, P < 0.01).

These results suggest that differences in 'Group' and 'Hemisphere' significantly influence eNOS expression, and the interaction between these factors and 'Dissected Region' further affects the patterns of eNOS expression, which provides an insight into the complex interplay of physiological conditions on eNOS activity in different brain regions.

In our analysis, multiple significant differences were observed across comparisons, as indicated in Fig. 6B[left]. Hemispheres treated with KCl, with or without cooling, showed significant changes in tubulin expression compared to naive hemispheres. These changes in expression indicate that KCl-induced SD explicitly affects the regulation of tubulin, an essential component of the neuronal cytoskeleton.

Furthermore, when correction coefficients were applied to the data, the observed trend in tubulin expression changes remained consistent (Fig. 6B [right]). A 3-way ANOVA revealed significant main effects for 'Group' (F(1, 60) = 31.78, P < 0.01) and a significant main effect for 'Hemisphere' (F(1, 60) = 22.48, P < 0.01). Regarding interactions, there was a significant 'Group' * 'Dissected region' interaction (F(2, 60) = 14.70, P <.0001). Additionally, a significant 'Group' * 'Hemisphere' interaction was observed (F(1, 60) = 8.56, P < 0.01). Finally, the analysis showed a significant three-way interaction ('Group' * 'Hemisphere' * 'Dissected region'; F(2, 60) = 13.35, P < 0.01).

These results suggest complex interactions between 'Group,' 'Hemisphere,' and 'Dissected region' in their influence on the measured outcome. In the post-hoc test, even after correcting for cooling effects, changes in tubulin expression in the exposed hemisphere differed in some regions compared to the naive hemisphere. This consistency suggests that the effect on tubulin by KCl-induced SD is not an artifact, reinforcing the concern of the present study.

#### eNOS/tubulin in the KCl study

Since tubulin variation directly affects eNOS analysis, a 2-way ANOVA was performed to examine the effects of 'Group' and 'Hemisphere' only on the central region, where tubulin variation was minimal, regardless of corrections (Fig. 6 C). The analysis revealed a significant main effect for 'Group' (F(1,20)=5.08, P < 0.05), indicating differences in eNOS/tubulin as well as a significant interaction between 'Group' and 'Hemisphere' (F(1,20)=13.64, P < 0.01). Subsequent post-hoc analyses showed significant differences in eNOS/tubulin between the CL group in the left central region compared to the right central region (P < 0.05) and between the CL group in the right central region and the NC group in the right central region (P < 0.01).

Further analysis of corrected data using the 2-way ANOVA revealed a significant interaction between 'Group' and 'Hemisphere' (F(1,20)=17.03, P < 0.01). Moreover, post-hoc comparisons indicated significant differences between the CL group in the left central region and the NC group in the left central region (P < 0.05), between the left central region and the right central region within the CL group (P < 0.05), and between the left central and right central regions within the NC group (P < 0.05).

Compared to the naive side of the reaction without temperature control, the KCl-induced SD during temperature control suggests that warming promotes eNOS expression and cooling suppresses eNOS expression.

#### p-eNOS/eNOS in the KCl study

The cooling effect was observed to change in eNOS, but the effect could not be confirmed at the p-eNOS level (Fig. 6D). A three-way ANOVA was performed since it was unnecessary to account for tubulin variability in the analysis of p-eNOS/eNOS. The results showed a significant interaction among 'Group,' 'Hemisphere,' and 'Dissected region' (F(2, 60) = 3.95, P < 0.05). Post-hoc comparisons showed no significant changes (P > 0.1), which raises intriguing questions about other potential regulatory pathways or compensatory mechanisms that could maintain p-eNOS levels.

## Discussion

The present study was designed to determine the effects of FBC on SD. KCl-induced SD and related events, which cause numerous physiological responses on the brain surface, recorded using multimodal recording techniques. FBC at 15°C gradually reduced the frequency of SD occurrence per unit of time by preventing the retention of morphological patterns or trails of SD. However, SD was not restored even after rewarming. In addition, although p-eNOS, which directly contributes to NO production, was not affected by cooling, a decrease in the amount of stored eNOS was observed upon cooling.

### Effects of FBC on multimodal signals

In the present experiment, assuming an optimal cooling temperature of 15°C, FBC caused three phenomena in healthy brain tissue that were different from previous findings before the administration of KCl (Fig. 4). First, AC-ECoG was strongly suppressed by cooling at 15°C (Fig. 4). Our previous study showed that cooling at <10°C strongly suppressed the baseline or functional neuronal activity in awake animals while cooling at 15°C efficiently and selectively suppressed epileptiform discharges (Fujii et al., 2012). The present experiment used direct perfusion of cold saline solution as the cooling method. Therefore, cooling at 15°C may have more potent effects in this study than those in previous reports; although the anesthesia may have had some effect.

Second, the baseline DC-ECoG was transiently shifted to negative immediately upon cooling (Fig. 4). The DC-shift induced by cooling fundamentally represents depolarization of the cell membrane through dramatic decrease in potassium conductance with cooling. In addition, Lehmenkuhler (1990) observed that rapid cooling of the cortex from 36°C to 6–7°C induced SD. These SDs, like "normal" SDs, were accompanied by a very steep depolarization phase, but cooling to 14°C did not appear to induce SDs. In the present study, cooling was maintained stable at about 15°C without severe overshooting, but it remains possible that the rapid temperature drop induced SD. Future studies would benefit from examining the specific effects of different cooling velocities. In contrast, sodium conductance is maintained by the depolarization block (Volgushev et al., 2000). Nevertheless, based on the present results, we suppose that conductance may recover if cooling is applied for a longer period.

Third, CBF temporarily increased at the start of cooling. Rapid local cooling of non-glabrous skin without functional sympathetic nerves causes initial vasodilation followed by vasoconstriction (Faber, 1988, Yamazaki et al., 2006). The possible mechanism for the initial vasodilation is the non-adrenergic response and the direct effects of rapid cooling on myogenic tone as postulated from studies of pulmonary arterial and aortic smooth muscle preparations (Mustafa and Thulesius, 2001). Subsequent slow blood flow reduction influences the Rho–Rho kinase system (Johnson, 2007, Thompson-Torgerson et al., 2007). Additionally, a previous study reported on a single-point measurement using a laser Doppler flow meter; hence, two-dimensional measurements using a laser speckle flow meter may have picked up other phenomena such as congestion of the venous component.

As mentioned above, FBC has effects that differ from those in our previous studies. The optimal cooling temperature and methods require individual consideration for each disease or condition. However, we could not determine the optimal cooling temperature and duration in this experiment. Nevertheless, we could isolate the effects of cooling on normal brain activity before examining its impact on SD.

### Changes in KCl-induced SD and related events by FBC

FBC decreased the number of KCl-induced SD and related events at each period of KCl administration, which was directly related to the decrease in the velocity and duration of SD and SH.

Several factors may explain why cooling suppresses SD. It was reported that the reduction of BrT induced by hypothermia reduces the occurrence of SD (Yenari et al., 2000). Our previous intraoperative human study showed that FBC strongly reduced not only epileptic discharges but also glutamate and lactate concentrations in epileptic foci (Nomura et al., 2014). The early induction of brain hypothermia facilitates faster recovery of cerebral perfusion pressure, repolarization, and the suppression of excessive glutamate release (Murai et al., 2022). Hinzman et al. (2016) reported that glutamate plays a critical role in triggering epileptic seizures and SD. Hence, the suppression of glutamate release due to FBC can suppress SD generation.

It is also possible that cooling prevents the deterioration of pathological conditions by inhibiting SD. These results seem consistent with those of other studies, which found that chronic systemic treatment with a prophylactic migraine drug reduced the number of SD events; however, the strength of inhibition depends on the treatment duration (Ayata et al., 2006). Remarkably, the expression of several DC-shifts is an essential indicator of physiological deterioration, such as excitotoxicity and metabolic crisis, and shows duration-dependent increases in glutamate and lactate levels and lactate/pyruvate ratio and causing SD in the peri-lesional cortex of patients with traumatic brain injury (Dreier et al., 2017, Hinzman et al., 2016).

It should be noted that the number of SDs has decreased, but the duration has extended. Bureš et al. (1974) argued that while the amplitude of electrical activity is influenced by the electrochemical equilibrium across the cellular membrane, the spreading velocity and the duration of depolarization are determined by more complex processes regulating membrane permeability. They noted that changes in potassium diffusion coefficient alone do not fully account for these observations. As evidenced by the fact that action potential firing rates are strongly suppressed at low temperatures (Nomura et al., 2019), suggesting reduced activity of Na^+^-K^+^ pumps in neurons and endothelial cells as one potential mechanism (de la Peña et al., 2012, Kang et al., 2005, Schneider et al., 2014). Additionally, the reported decreased activity of K^+^ pumps in glial cells, as seen in hibernating animals (Nikolic et al., 2014), indicates that cooling may affect the homeostasis of intracellular and extracellular ion concentrations, potentially delaying the stabilization of brain states. Although the negative impacts of an extended SD duration, possibly due to these mechanisms, have not been fully elucidated, supported arguments are as follows. Prolonged or long-lasting SD, which indicates duration longer than that observed in an intact, normally perfused brain, suggests some degree of dysfunction or metabolic compromise that delays repolarization (Hartings et al., 2017). The duration of the negativity is a measure of the local metabolic and excitotoxic burden imposed on tissue by SD (Dreier et al., 2017). There are, however, other possible explanations. Cooling has both neuroprotective effects and suppresses neural activity, which alters the threshold for metabolic disturbance, even though, at first glance, cooling appears to transition the brain to a state more susceptible to metabolic disturbance. Thus, although cooling may prolong the duration of SD by inhibiting neural activity, reducing neurotransmitter release, and diminishing ion concentration regulation, it may also stabilize brain states and alleviate the pathological processes that trigger SD.

This experiment required some considerations regarding the cooling temperature and time. Since a cooling temperature of 15°C was found to suppress epileptic seizures (Fujii et al., 2012), we thought it could also be effective for SD. At 15°C, the appearance of SD gradually disappeared. Although the cooling time was long compared to previous studies of FBC (He et al., 2019, Inoue et al., 2017), the lowest cooling temperature and cooling time was 1 h at 0°C (Oku et al., 2009), which we assumed the brain would tolerate. However, this was not the case. Since cooling can cause increased BBB permeability(Inamura et al., 2013) and decreased energy metabolism (Nomura et al., 2014), this may act as a neuronal and vascular function stressor. Future studies should examine whether SD is suppressed at higher cooling temperature ranges, for example, cooling at 25–30°C. This investigation may determine the optimal FBC temperature that minimizes stress on the brain parenchyma.

### eNOS production modulation by FBC

While the stability of housekeeping proteins is crucial for Western blot analysis, the tubulin employed in this study was affected by low temperature. Low temperatures generally reduce enzymatic activity and slow biochemical reactions. Processes such as protein synthesis and modification may be affected as well. Hence, cooling may alter the dynamics of microtubules containing tubulin. Indeed, using yeast cells as a model, studies examining how low temperature affects microtubule dynamics have shown that low temperature decreases the rate of tubulin polymerization and depolymerization, ultimately causing a rapid decrease in microtubule polymers (Li and Moore, 2020). Further, human iPSC-derived neurons undergo a decrease in tubulin protein levels upon exposure to low temperatures, coinciding with the loss of microtubule polymers (Ou et al., 2018). These findings raise concerns about the reliability of tubulin as a loading control, as they could lead to misleading interpretations of our results, especially those related to eNOS expression. One limitation of this study is the evaluation of eNOS with tubulin as a control, as cooling may influence tubulin. In future research, it will be necessary to employ a substance unaffected by temperature as a control. One limitation of this study is the evaluation of eNOS with tubulin as a control, as cooling may influence tubulin. In future research, it will be necessary to employ a substance unaffected by temperature as a control. Additionally, it may be necessary to assess the absolute levels of eNOS independently of tubulin normalization to verify the effects of cooling more accurately.

Despite the observed sustained suppression of SD, no significant increase or decrease in p-eNOS/eNOS, indicating eNOS activation, was observed in both NC and CL groups. In contrast, cooling affected eNOS reserves the following: the NC group showed an increase in eNOS, while the CL group showed a significant reduction in eNOS. These changes may have been because brain tissue was collected immediately after SD induction. Since prolonged cooling had a more substantial effect on brain tissue, brain tissue was collected immediately after SD induction to avoid the adverse effects of prolonged exposure to FBCs on the brain parenchyma in Western blot analysis. Therefore, the lack of significant changes in p-eNOS expression in the CL group is likely due to the short time between SD induction and tissue collection, reflecting an initial response to SD in the FBC rather than the effects of repeated or prolonged SD.

eNOS/β-tubulin expression was decreased by FBC at 15°C at the central region. We think that these results are reasonable even if KCl administration increases eNOS expression. According to a previous study, thermal control can alter the expression level of eNOS. Using human microvascular endothelial cells and bovine aorta endothelial cells, Binti Md Isa et al. (2011) suggested that the combination of shear stress with thermal changes affect eNOS expression; thermal control at 37°C and 4°C increases and decreases eNOS activation, respectively. Although the mechanisms of the rapid change in eNOS expression remains to be established, the potential mechanisms may include proteolysis, calcium-dependent turnover (Govers and Rabelink, 2001), and changes in eNOS mRNA stability (Fleming and Busse, 2003). Previous studies have indicated that factors such as tumor necrosis factor-α and vascular endothelial growth factor may modulate eNOS expression (Fleming and Busse, 2003). Although not investigated in the current study, future research should focus on these factors as potential regulators in the context of FBC.

The suppression of eNOS by FBC may have an adverse effect on SDs that occurs after subarachnoid hemorrhage. Earlier studies demonstrated that NO alters the threshold for depolarization, which is mainly maintained by eNOS; thus, increasing NO availability may be beneficial in patients with subarachnoid hemorrhage. (Petzold et al., 2008). Unfortunately, cooling was not involved in increasing the production efficiency of NO in the present results. However, FBC was able to prevent SD as an electrical phenomenon, which may inhibit its effect on subsequent vasoreactivity. Besides, one study showed that the increase in focal brain injury-induced neuronal NOS expression, which is one of the vasodilation factors of SD, was suppressed by moderate hypothermia (Miyake et al., 1999). While we did not perform immunohistochemistry in this study, such an approach could provide more localized and detailed insights into eNOS expression changes, increasing the understanding to how FBC affects eNOS at the tissue level.

In summary, our findings suggest a potential link between FBC and the suppression of eNOS expression, although the underlying mechanisms remain unclear. Future research should focus on the role of proteolytic pathways and explore the influence of other factors like tumor necrosis factor-α and vascular endothelial growth factor. Such comprehensive research will be critical for a deeper understanding of the neurovascular dynamics underlying FBC.

### The possibility of using thermal neuromodulation and multimodality monitoring for neurological and neurosurgical disease

Recently, non-invasive neuromodulation has been recognized as a valuable and valid strategy for migraine management when traditional pharmacological treatments are insufficient (Dahlem, 2013). If thermal neuromodulation inhibits SD which has been widely accepted as the electrophysiological substrate of migraine aura and a putative headache trigger (Ayata, 2010), this method might become another promising tool for migraine management. However, it is necessary to simplify the device and lower the psychological threshold for implantation. We anticipate that applications of FBC for SD will be practical for more severe neurosurgical diseases. FBC can be temporarily utilized to prevent the development of SD in the perioperative management of severe neurosurgical diseases such as cerebral infarction, subarachnoid hemorrhage, and traumatic brain injury (COSBID study group, 2012, Lauritzen et al., 2011). As delayed cerebral ischemia after subarachnoid hemorrhage, which correlates with the frequency of SD, significantly impacts patient outcomes, suppressing the frequency of SD with FBC may lead to the suppression of delayed cerebral ischemia, especially if SD causes delayed cerebral ischemia.

In the acute phase of such diseases, especially in cases of severe injury, whole-body hypothermia may be considered as a treatment option. However, it is essential to carefully evaluate the potential for adverse events, including immune suppression and deficiency of coagulation. By contrast, for scenarios requiring surgical interventions, such as decompressive craniotomy, implementing an intracranial FBC device to directly cool the injured site could be a safer alternative. This procedure allows the FBC to manage symptoms and potentially reduce damage in acute neurologic disease but should always be monitored by testing effects such as ischemic expansion by eNOS suppression and, if necessary, terminate cooling at a higher cooling temperature. It should be noted, however, the effect of SD is controversial (Sugimoto et al., 2023). Besides, suppression of eNOS by cooling may hurt conditions in which nitric oxide plays a neuroprotective role, such as stroke or traumatic brain injury.

Further studies are needed to elucidate the effect of SD and to introduce thermal neuromodulation of SD. This study produced results that corroborate the findings of many of the multimodal measurements of SD. Multimodal measurement is especially meaningful when single modality sensing cannot capture signals due to the sensing accuracy and neuromodulation effects. In the present study, separate devices were used to measure each modality, with the expectation that in the future, a multimodal sensor that combines all sensing elements necessary into a single device will be developed; research and development are underway.

## Conclusions

Using multimodal recording techniques provides relatively accurate results, and cooling the brain surface can effectively reduce KCl-induced SD, SH, and spreading depression, in a time-dependent manner. These findings have significant implications for understanding the regulation of pathogenic conditions through brain cooling. Furthermore, FBC is expected to regulate SD by combining the modulation of neural cell activity and eNOS expression, serving as an approach to adjust SD. Additionally, establishing the optimal time interval and temperature for FBC may mitigate migraine or ischemia on brain tissue by regulating SD. Nevertheless, further research is needed to determine the optimal duration and temperature of FBC to achieve the most favorable results and to ensure its safety and efficacy in modulating SD.

## Funding

This work is supported by 10.13039/501100001691JSPS KAKENHI [grant numbers 15H05719, 16K15646, 22K16661] and 10.13039/501100001695JST FOREST [grant number JPMJFR2152].

## CRediT authorship contribution statement

**Hirochika Imoto:** Conceptualization, Methodology. **Sadahiro Nomura:** Writing – review & editing. **Satoshi Shirao:** Conceptualization, Methodology. **Yuya Hirayama:** Formal analysis, Investigation, Visualization. **Michiyasu Suzuki:** Funding acquisition, Writing – review & editing. **Kazutaka Sugimoto:** Conceptualization, Methodology, Writing – review & editing. **Fumiaki Oka:** Conceptualization, Methodology, Writing – review & editing. **Hiroyuki Kida:** Formal analysis, Investigation, Methodology, Visualization, Writing – review & editing. **Takao Inoue:** Conceptualization, Methodology, Writing – original draft, Funding acquisition.

## Declaration of Competing Interest

The authors declare that they have no conflicts of interest.
