# Supplementary material for: Focal brain cooling suppresses spreading depolarization and reduces endothelial nitric oxide synthase expression in rats
Source: IBRO Neurosci Rep. 2024 May 12;16:609–21. doi: 10.1016/j.ibneur.2024.05.001 (PMC11127172; doi:10.1016/j.ibneur.2024.05.001)
Supplement: Supplementary file 3 — Supplementary material [file mmc1.pptx]

## Slide 1
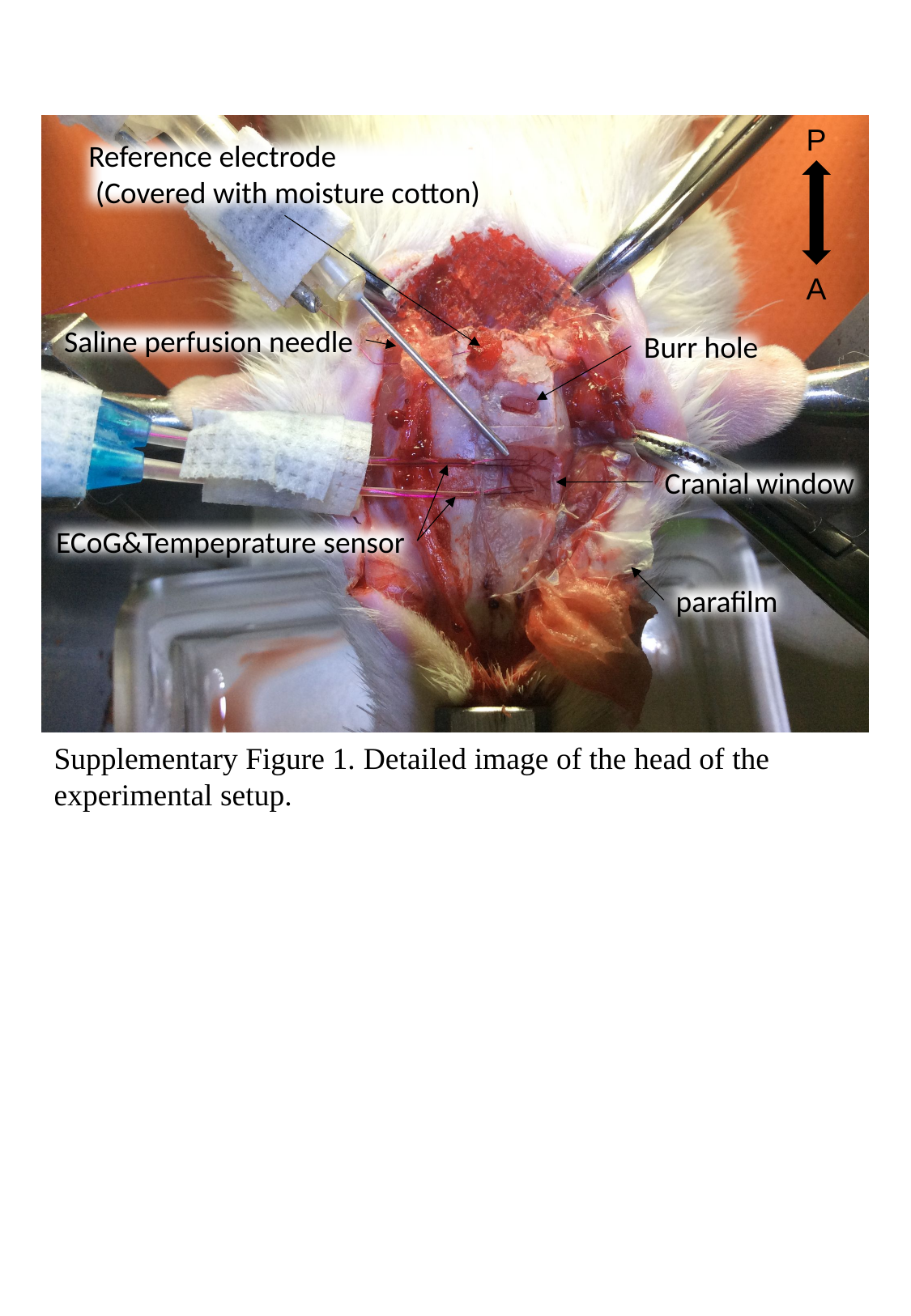

P
Reference electrode
 (Covered with moisture cotton)
A
Saline perfusion needle
Burr hole
Cranial window
ECoG&Tempeprature sensor
parafilm
Supplementary Figure 1. Detailed image of the head of the experimental setup.

## Slide 2
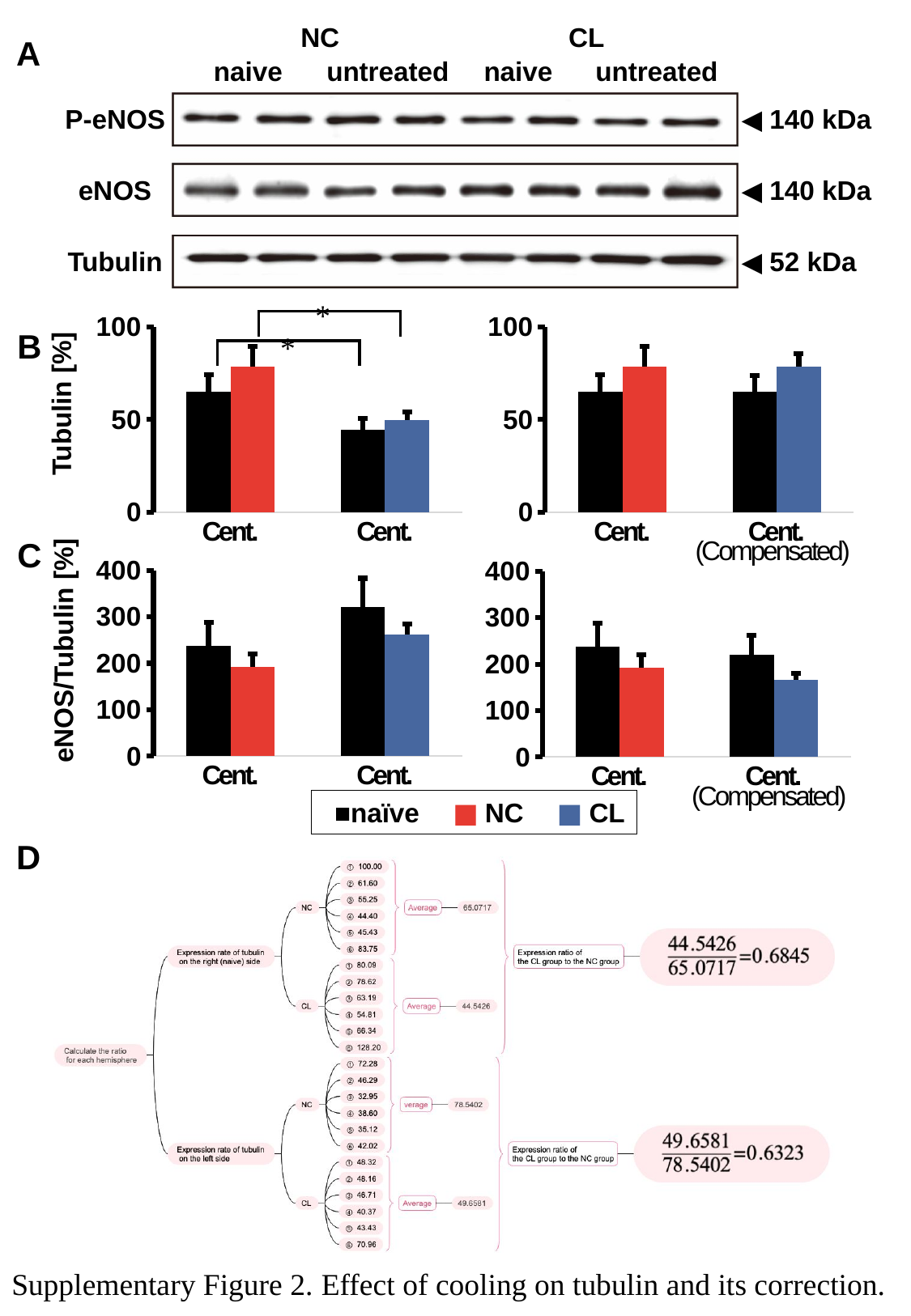

NC
CL
A
naive
untreated
naive
untreated
P-eNOS
◀︎ 140 kDa
eNOS
◀︎ 140 kDa
Tubulin
◀︎ 52 kDa
*
### Chart
| Category | contra- | ipsi- |
|---|---|---|
| Cent. | 65.07 | 78.54 |
| Cent. | 44.54 | 49.66 |
### Chart
| Category | contra- | ipsi- |
|---|---|---|
| Cent. | 65.07 | 78.54 |
| Cent. | 65.07 | 78.54 |B
Tubulin [%]
*
eNOS/Tubulin [%]
(Compensated)
C
### Chart
| Category | contra- | ipsi- |
|---|---|---|
| Cent. | 238.3 | 192.54 |
| Cent. | 321.1 | 262.92 |
### Chart
| Category | contra- | ipsi- |
|---|---|---|
| Cent. | 238.3 | 192.54 |
| Cent. | 219.8 | 166.24 |(Compensated)
■naïve　■NC　■CL
D
Supplementary Figure 2. Effect of cooling on tubulin and its correction.

## Slide 3
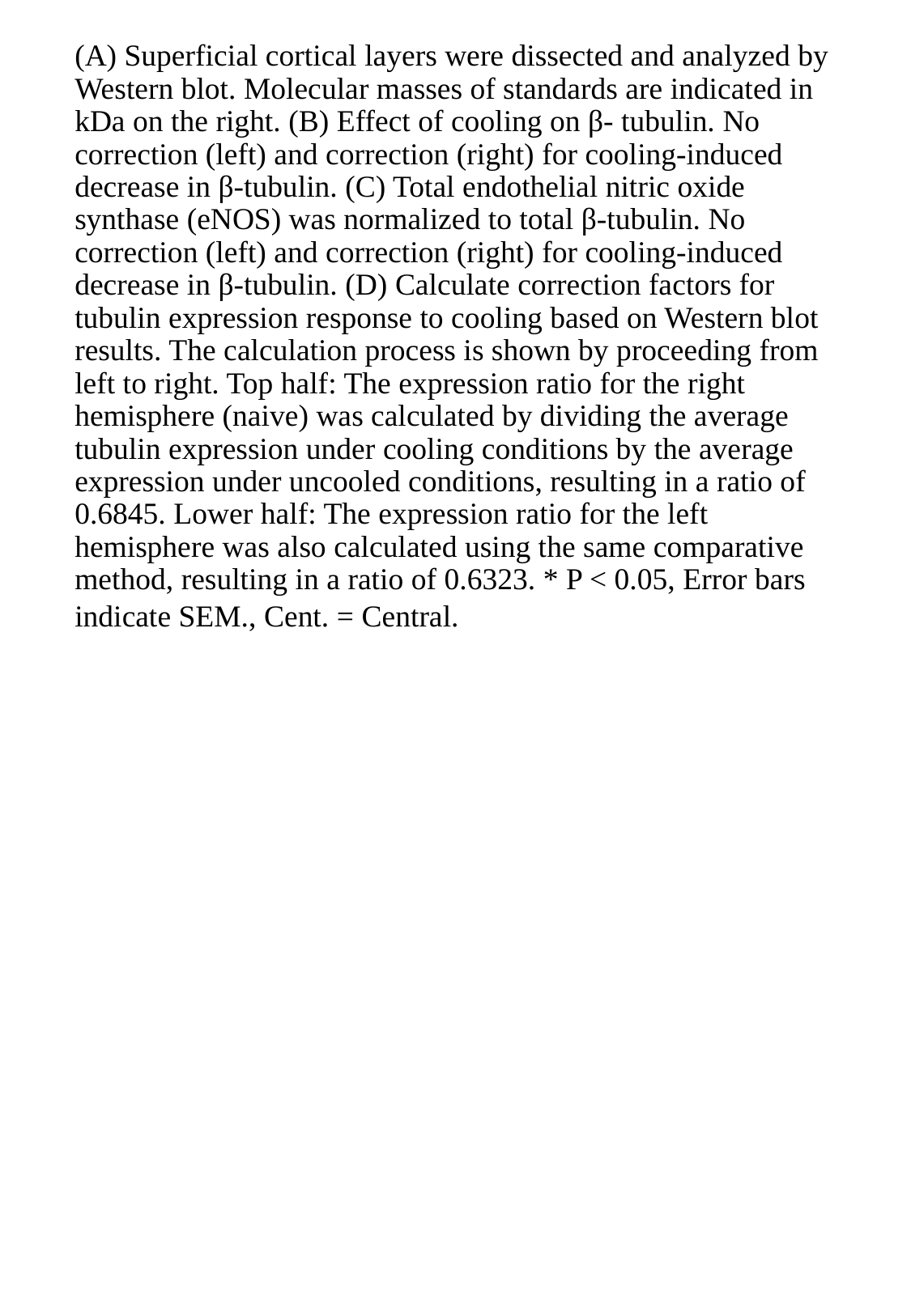

(A) Superficial cortical layers were dissected and analyzed by Western blot. Molecular masses of standards are indicated in kDa on the right. (B) Effect of cooling on β- tubulin. No correction (left) and correction (right) for cooling-induced decrease in β-tubulin. (C) Total endothelial nitric oxide synthase (eNOS) was normalized to total β-tubulin. No correction (left) and correction (right) for cooling-induced decrease in β-tubulin. (D) Calculate correction factors for tubulin expression response to cooling based on Western blot results. The calculation process is shown by proceeding from left to right. Top half: The expression ratio for the right hemisphere (naive) was calculated by dividing the average tubulin expression under cooling conditions by the average expression under uncooled conditions, resulting in a ratio of 0.6845. Lower half: The expression ratio for the left hemisphere was also calculated using the same comparative method, resulting in a ratio of 0.6323. * P < 0.05, Error bars indicate SEM., Cent. = Central.

## Slide 4
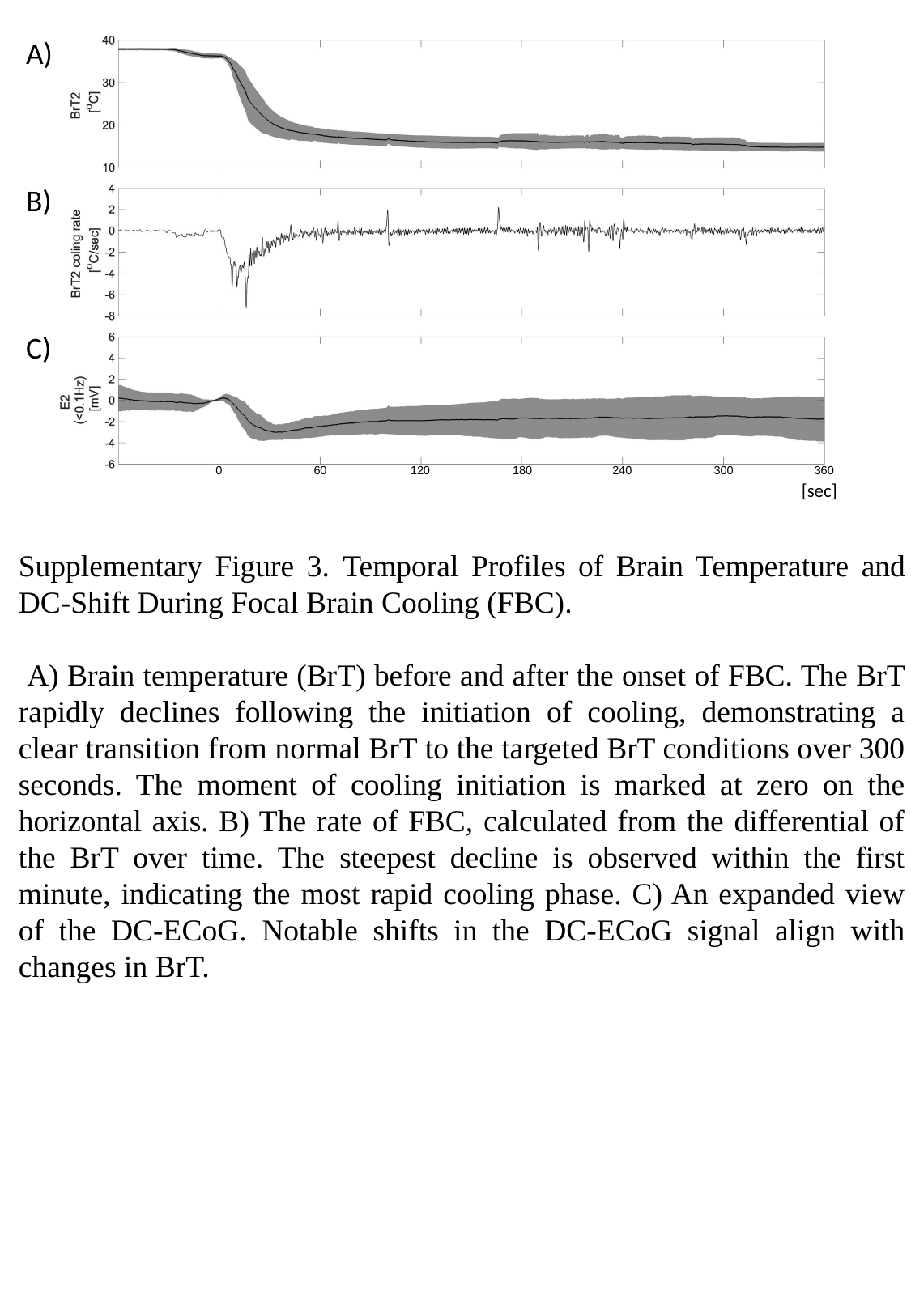

A)
B)
C)
0
60
120
180
240
300
360
[sec]
Supplementary Figure 3. Temporal Profiles of Brain Temperature and DC-Shift During Focal Brain Cooling (FBC).
 A) Brain temperature (BrT) before and after the onset of FBC. The BrT rapidly declines following the initiation of cooling, demonstrating a clear transition from normal BrT to the targeted BrT conditions over 300 seconds. The moment of cooling initiation is marked at zero on the horizontal axis. B) The rate of FBC, calculated from the differential of the BrT over time. The steepest decline is observed within the first minute, indicating the most rapid cooling phase. C) An expanded view of the DC-ECoG. Notable shifts in the DC-ECoG signal align with changes in BrT.
